# Early Screening of Autism among 18 to 24 months Old Toddlers Using the Quantitative Checklist for Autism in Toddlers (Q-CHAT)

**Published:** 2020

**Authors:** Mohammad Reza MOHAMMADI, Ghazal ZAHED, Hadi ZARAFSHAN

**Affiliations:** 1 Department of Autism and Neurodevelopmental Disorders, Psychiatry and Psychology Research Center, Tehran University of Medical Sciences, Roozbeh Psychiatric Hospital,Tehran, Iran.; 2 Child and Adolescent psychiatrist Assistant Professer of Shahid Beheshti University of Medical Sciences, Mofid Childrens Hospital, Tehran, Iran.

**Keywords:** Autism, Early Screening, Q-CHAT, Toddler

## Abstract

**Objective:**

The aim of the present study was to screen the toddlers who are suspected to be autistic in their well-child visits at age 18 to 24 months via the Quantitative-Checklist for Autism in Toddlers (Q-CHAT).

**Materials & Methods:**

After the screening, the screen-positive cases were clinically assessed by a child psychiatrist and a child developmental psychologist. The total sample included 2467 toddlers; the mean total score of Q-CHAT was 30.64 (SD: 9.133).

**Result:**

According to the clinical examinations, 6 cases met the criteria for autism (equal to 5.8% of screen-positive cases and 0.25% of the total sample), and 18 cases had a high probability of autism or other neurodevelopmental disorders.

**Conclusion:**

This study showed that the toddlers who were at risk of autism could be screened in PHC. Also, Q-CHAT could be used as a screening tool in Iran.

Autism is a neurodevelopmental disorder that affects the development of children from the outset (1). It is characterized with deficits in social and communication skills and restricted and repetitive behaviors that usually appear around the first birthday (1, 2).

It is necessary to conduct early screening and diagnosis in the autistic children since it can improve the subsequent outcomes (3, 4). Most early screening programs have been implemented in high-income countries, especially in the US [5]. In recent years, some attempts have been made to establish early screening programs in health care systems of low- and middle-income countries (LMICs) (6-8).

Similar to many other LMICs, Iran is a novice country in the early screening of autism and other neurodevelopmental disorders. In recent years, some epidemiologic studies have focused on screening autism among Iranian children older than 2 years in different settings using different methods (9-11). 

The literature has emphasized on detecting the children who are at risk of autism when they are 18 to 24 months old (2). It suggests that primary care is a good setting for early screening programs that can reduce the age at diagnosis (12). Accordingly, the present study aimed to design and investigate an early screening program in primary health care (PHC) system of Iran.

In the new edition of the Diagnostic and Statistical Manual of Mental Disorders ( DSM-5), the definition of autism has changed from a categorical disorder with different subgroups to a dimensional diagnostic category (10). Various screening and diagnostic tools have been developed to treat the symptoms of autism as a continuous concept rather than a category. (13). One of these tools is the Quantitative Checklist for Autism in Toddlers (Q-CHAT), which has recently gained attention as a level 1 screening tool for toddlers. It is a 25-item parent-report screening tool for autistic symptoms for toddlers aged 18–24 months. It measures developmentally relevant traits and behaviors associated with autism [14]. The reliability and predictive validity of the Persian version of Q-CHAT have been investigated previously in a clinically referred samples of toddlers. The present study showed that the Persian version of Q-CHAT can distinguish toddlers who are at risk of autism from those who are typically developing [15]. In the next step, this study aimed to investigate the feasibility of using Q-CHAT to screen toddlers who are suspected to be autistic at age 18 to 24 months in their well-child visits in the primary care setting of Iran.

## Materials & Methods

Participants and Setting:

This population-based study aimed to investigate the feasibility of screening toddlers who are at risk of autism in the PHC setting as early as possible in Iran. The primary health care (PHC) system of Iran is distributed throughout all urban and rural areas of the country. The “Health Posts” and “Health Houses” are the first point of contact of PHC in urban and rural areas, respectively. Urban and rural health centers are the next level of care with services for more complex problems. This network is managed and administered by district health centers, which are under the supervision of universities of medical sciences in each province.

Tehran, the capital of Iran, was selected for this study. With a population of more than 8 million, Tehran is the most populous city in Iran whose population is a combination of all Iranian ethnic groups. Due to its large size, Tehran was divided into 3 districts, each of which was covered by Tehran University of Medical Sciences, Iran University of Medical Sciences, and Shahid Beheshti University of Medical Sciences. We selected the urban districts (7 districts) covered by Tehran University of Medical Sciences with a population of near 2 million.

Based on the statistical formula, the sample size of 2401 was estimated for this population-based study, and 10% was added to this sample to control any missing data (N = 2641). This study included all the toddlers who were brought by their family for 18-24-month visits and vaccinations to Health Posts located in the selected districts.

Outcome Measures:

Demographic Questionnaire:

Demographic questionnaire consisted of a number of questions about child development, medical history, and information about parental education, medical and job history. 

The Quantitative Checklist for Autism in Toddlers (Q-CHAT):

The Q-CHAT [14] is a 25-item caregiver-report screening measure of autistic traits for toddlers aged 18–24 months. The items are rated on a 5-point Likert scale (0–4) with higher ratings indicating more autistic traits. Thirteen items are reversely scored. The Persian version of Q-CHAT has good psychometric properties. The Cronbach’s alpha coefficient of the checklist was 0.886, and test-retest reliability was calculated as 0.997 (p<0.01). The estimated area under the curve (AUC) was 0.971[15].

Procedure:

After receiving the legal permissions and ethical code from Tehran University of Medical Sciences, all Health Posts located in the selected districts were contacted. In sum, 20 of them, which were distributed throughout the districts, agreed to participate in this study. In each health posts, one person from the Family Health Section was selected as the contact person and coordinator. Then, they were informed about the aim of the study and the items of the demographic questionnaire and Q-CHAT.

When families came for appointments and vaccinations for their 18- to 24-month-old children, the coordinator explained the purpose of the project and the parents' rights based on the informed consent form and asked the parents to sign the consent form if they agreed. Then, the coordinator asked them to fill out the questionnaires anonymously. This process was continued until the expected sample size was achieved.

If the score in Q-CHAT was more than expected (total score   35), the family would be sent to the Psychiatry and Psychology Research Center (PPRC) of Tehran University of Medical Sciences in Roozbeh psychiatric hospital for additional investigation. Parents needed to make an appointment to visit a psychiatrist. At PPRC, a child and adolescent psychiatrist and a child developmental psychologist interviewed families and observed their children based on DSM-5 criteria for autism. 

Data analysis:

Data were analyzed using IBM SPSS Statistics version 23.0.

## Results

In sum, a total of 2704 questionnaires were filled out, 138 of which were incomplete. In 99 questionnaires, the children were younger than 17 months, thus, the total sample size was reduced to 2467 toddlers. The participants' age range was between 17 to 27 months (Mean: 19.6, SD: 2.48), 48.7% of whom were female, and 51.3% were male. Also, 54.1% were the first child, 30% were vaginally delivered, and 70% were delivered by cesarean section. The age range of fathers was 23-60 years (Mean 35.53, SD: 5.75) and that of mothers was 19-45 years (Mean 31.02, SD: 4.1).

The range of the total score of Q-CHAT among participants was 11-72 (Mean 30.64, SD: 9.133) (Table 1).

**Table 1:** Distribution of the Total Scores of All Participants in Q-CHAT

Total score	Frequencies	Percent	Cumulative Percent	Total score	Frequencies	Percent	Cumulative percent
11.00	13	.5	.5	35.00	94	3.8	69.2
12.00	4	.2	.7	36.00	118	4.8	74.0
13.00	16	.6	1.3	37.00	108	4.4	78.4
14.00	28	1.1	2.5	38.00	74	3.0	81.4
15.00	39	1.6	4.1	39.00	74	3.0	84.4
16.00	40	1.6	5.7	40.00	60	2.4	86.8
17.00	39	1.6	7.3	41.00	52	2.1	88.9
18.00	50	2.0	9.3	42.00	23	.9	89.9
19.00	44	1.8	11.1	43.00	45	1.8	91.7
20.00	59	2.4	13.5	44.00	18	.7	92.4
21.00	96	3.9	17.3	45.00	29	1.2	93.6
22.00	73	3.0	20.3	46.00	52	2.1	95.7
23.00	107	4.3	24.6	47.00	25	1.0	96.7
24.00	60	2.4	27.1	48.00	22	.9	97.6
25.00	71	2.9	30.0	49.00	15	.6	98.2
26.00	90	3.6	33.6	50.00	15	.6	98.8
27.00	80	3.2	36.8	51.00	4	.2	99.0
28.00	134	5.4	42.3	52.00	5	.2	99.2
29.00	99	4.0	46.3	53.00	4	.2	99.4
30.00	147	6.0	52.2	54.00	7	.3	99.6
31.00	74	3.0	55.2	69.00	1	.0	99.7
32.00	78	3.2	58.4	70.00	3	.1	99.8
33.00	69	2.8	61.2	71.00	3	.1	99.9
34.00	104	4.2	65.4	72.00	2	.1	100.0

Based on the total Q-CHAT score, more than 35853 cases (29.8%) were screened positive and referred to PPRC. However, only 104 (12%) made an appointment. The mean age of those toddlers whose parents made an appointment for a psychiatric visit was 19.63 months (sd: 2.6), and 38.5% were female, and 61.5% were male. The mean of the total Q-CHAT score was 42.98 (sd: 7.27) and ranged from 35 to 72 (Table 2).

**Table 2:** Distribution of the Total Scores of Positively Screened Participants in Q-CHAT

Total score	Frequencies	Percent	Cumulative percent	Total score	Frequencies	Percent	Cumulative percent
35.00	6	5.8	5.8	47.00	4	3.8	82.7
36.00	7	6.7	12.5	48.00	5	4.8	87.5
37.00	9	8.7	21.2	49.00	2	1.9	89.4
38.00	8	7.7	28.8	50.00	2	1.9	91.3
39.00	9	8.7	37.5	51.00	1	1.0	92.3
40.00	6	5.8	43.3	52.00	2	1.9	94.2
41.00	6	5.8	49.0	53.00	1	1.0	95.2
42.00	6	5.8	54.8	54.00	1	1.0	96.2
43.00	9	8.7	63.5	70.00	2	1.9	98.1
44.00	4	3.8	67.3	71.00	1	1.0	99.0
45.00	5	4.8	72.1	72.00	1	1.0	100.0
46.00	7	6.7	78.8				

The clinical evaluation, based on DSM-5 criteria and interviews with parents regarding toddlers' developmental and medical history, showed that 6 cases met the criteria for autism (equal to 5.8% of screen-positive cases and 0.25% of the total sample). Also, 18 cases were at high risk of autism or other neurodevelopmental disorders (equal to 17.3% of screen-positive cases and 0.73% of the total sample).

The mean of the total Q-CHAT score was 64.83 among autistic cases (sd: 9.21) and ranged 52 to 72. Also, this mean was 48.22 (sd:2.4) among high probability cases and ranged 44 to 53.

## Discussion

This was the first study to screen autism before the age of 24 months in Iran using a screening tool (Q-CHAT), which considers the symptoms of autism as a continuous concept rather than a categorical one.

The mean of the total score of Q-CHAT among the participants was 30.64 (SD: 9.133). In another population-based study in Singapore, using-CHAT, the mean score of 18 months old toddlers was 35.57 (SD:7.21) and it was 33.2 among 24 months olds (SD:7.7) [13]. In a previous study in Iran, the mean of the total score for the typically developing group was 22.4 (SD = 6.26) on Q-CHAT and 50.94 (SD =12.35) for the ASD group [15]. In the present study, the mean scores were higher than the previous study because, in the previous study, the mean age of participants (27.14 months) was higher than the present study and that younger toddlers were developmentally more immature, and thus typically receive more scores.

Based on the total Q-CHAT score, 29.8% of the toddlers in this sample were positive screen, which was much higher than expected for the first stage screening tool. According to the results of the present study, 5.8% of clinically evaluated cases, equivalent to 0.25% of the total sample, met the autism criteria (25 per 10,000).Given that none of the screen-positive cases were sent for clinical assessment (only 12%), it can be stated that this estimate of the prevalence of autism among Iranian toddlers should be considered with caution, as it is much lower than the suspected prevalence of autism. Based on the data of this study, the minimum score of the ASD group in Q-CHAT was 52. By considering this score in the total scores of the sample (Table 1), 0.8% of the participants obtained a score of more than 52, which is near to the accepted global prevalence of autism in other population-based studies. The other 17.3% of clinically assessed cases that were equal to 0.73% of the total sample were at high risk of autism or other neurodevelopmental disorders (73 per 10 000).

In another study conducted in Mahabad, Iran, the prevalence of autism and ASD among 2 and 3 year-olds was reported as 15 per 10 000 and 77 per 10 000, respectively [9]. 

## Limitations and Recommendations:

The most notable limitation of this study was the use of a standardized diagnostic tool. Considering that there are no standard diagnostic tools for this age group in Iran, it was decided to use the clinical interview based on DSM-5 criteria for diagnosis.

The total score of 35 in Q-CHAT was considered as the cutoff point, leading to a huge number of screen-positive cases (29.8%) compared to final suspected cases (0.25%) and a high false positive for this cutoff point. Thus, cutoff points with more reasonable screen positive or false positive should be used in future studies and in practice. According to Table 1, if the total score of 50 was considered as the cutoff point, about 12% of the cases would screen positive, which seems reasonable. The total number of 50 can be used as the cutoff point to screen toddlers who are at risk of autism in the Iranian population.

Another limitation of this study was the low rate of participation in clinical assessment sessions by families (12%), for which we used a coordinator in the screening system to track and coordinate people who need clinical evaluation. Also, conducting telephone interviews for screen-positive cases could help increase the number of cases that received diagnostic evaluation. 

## In Conclusion

In sum, the present study revealed that the toddlers who were at risk of autism in PHC in Iran could be screened via Q-CHAT as a level 1 screening tool in Iranian culture. However, standardized tools should be used to diagnose autism among very young children in Iran.

## References

[1] Association, A.P (2013). The Diagnostic and Statistical Manual of Mental Disorders: DSM 5.

[2] Johnson CP, SM Myers ( 2007). Identification and evaluation of children with autism spectrum disorders. Pediatrics.

[3] MacDonald R (2014). Assessing progress and outcome of early intensive behavioral intervention for toddlers with autism. Research in developmental disabilities.

[4] Orinstein A J (2014). Intervention for optimal outcome in children and adolescents with a history of autism. Journal of developmental and behavioral pediatrics: JDBP.

[5] Stewart L A (2017). Screening for autism spectrum disorder in low-and middle-income countries: A systematic review. Autism.

[6] Kakooza-Mwesige A (2014). Adaptation of the “ten questions” to screen for autism and other neurodevelopmental disorders in Uganda. Autism.

[7] Kara B (2014). Using the Modified Checklist for Autism in Toddlers in a well-child clinic in Turkey: Adapting the screening method based on culture and setting. Autism.

[8] Wang J (2012). Assessing autistic traits in a Taiwan preschool population: cross-cultural validation of the Social Responsiveness Scale (SRS). Journal of autism and developmental disorders.

[9] Samadi SA, R McConkey (2015). Screening for autism in Iranian preschoolers: Contrasting M-CHAT and a scale developed in Iran. Journal of autism and developmental disorders.

[10] Samadi SA (2016). The challenges of screening pre-school children for autism spectrum disorders in Iran. Disability and rehabilitation.

[11] Samadi S A, A Mahmoodizadeh (2012). A national study of the prevalence of autism among five-year-old children in Iran. Autism.

[12] Khowaja M, DL Robins (2017). Utilizing two-tiered screening for early detection of autism spectrum disorder. Autism.

[13] Magiati I (2015). The psychometric properties of the Quantitative-Checklist for Autism in Toddlers (Q-CHAT) as a measure of autistic traits in a community sample of Singaporean infants and toddlers. Molecular autism.

[14] Allison C (2008). The Q-CHAT (Quantitative CHecklist for Autism in Toddlers): A normally distributed quantitative measure of autistic traits at 18–24 months of age: Preliminary report. Journal of Autism and Developmental Disorders.

[15] Mohammadian M (2015). Evaluating Reliability and Predictive Validity of the Persian Translation of Quantitative Checklist for Autism in Toddlers (Q-CHAT). Iranian journal of psychiatry.

